# Optimized Proteome Reduction for Integrative Top–Down Proteomics

**DOI:** 10.3390/proteomes11010010

**Published:** 2023-03-06

**Authors:** Breyer Woodland, Aleksandar Necakov, Jens R. Coorssen

**Affiliations:** 1 Department of Biological Sciences, Faculty of Mathematics and Science, Brock University, St. Catharines, ON L2S 3A1, Canada; 2 Ronin Institute, Montclair, NJ 07043, USA

**Keywords:** dithiothreitol (DTT), 2-hydroxyethyl disulfide (HED), protein species, proteoform, reducing agent, tributylphosphine (TBP), two-dimensional gel electrophoresis (2DE)

## Abstract

Integrative top–down proteomics is an analytical approach that fully addresses the breadth and complexity needed for effective and routine assessment of proteomes. Nonetheless, any such assessments also require a rigorous review of methodology to ensure the deepest possible quantitative proteome analyses. Here, we establish an optimized general protocol for proteome extracts to improve the reduction of proteoforms and, thus, resolution in 2DE. Dithiothreitol (DTT), tributylphosphine (TBP), and 2-hydroxyethyldisulfide (HED), combined and alone, were tested in one-dimensional SDS-PAGE (1DE), prior to implementation into a full 2DE protocol. Prior to sample rehydration, reduction with 100 mM DTT + 5 mM TBP yielded increased spot counts, total signal, and spot circularity (i.e., decreased streaking) compared to other conditions and reduction protocols reported in the literature. The data indicate that many widely implemented reduction protocols are significantly ‘under-powered’ in terms of proteoform reduction and thus, limit the quality and depth of routine top–down proteomic analyses.

## 1. Introduction

Given their complexity and diversity, it is becoming far more widely recognized that analytical methods that do not effectively address the resolution of the full breadth of species (i.e., proteoforms) beyond primary amino acid sequences simply lack the depth required to provide an accurate, comprehensive characterization of proteomes [1,2,3,4]. Integrative top–down proteomics—tightly coupling two-dimensional gel electrophoresis (2DE; isoelectric focusing (IEF) followed by SDS-PAGE), liquid chromatography, and tandem mass spectrometry (LC/TMS), is currently the only available analytical approach that fully addresses the depth needed for effective and routine assessments of proteomes. In this regard, 2DE-based top–down proteomics has been widely employed for quantitative proteome analysis due to its well-established capacity to resolve many thousands of intact proteoforms from complex native proteome extracts [4,5,6,7,8,9,10]. Since its introduction, 2DE methodology has undergone rigorous review and assessment to improve resolution, detection/sensitivity, and thus, overall analytical rigour [11,12,13,14,15,16,17,18,19,20,21,22,23,24]. These continued refinements enable an even greater understanding of proteome complexity, thus increasing the depth of analyses with a routine focus on proteoforms rather than only canonical amino acid sequences. Integrative top–down proteomics is thus the only approach that currently enables deep proteome analyses (i.e., across a large range of pI and molecular weights) and, therefore, genuine understanding of molecular mechanisms and the identification of rational biomarkers and therapeutic targets.

Disulfide bonds between sulfhydryl groups of cysteine side chains often regulate proteoform folding and final structure. If not appropriately addressed, disulfide bridges can break and reform during 2DE, both intra- or intermolecularly. Such ‘scrambled bridges’ can result in additional, non-native species (i.e., ‘spots’) on a 2D map of a proteome [25]. Thus, an appropriate concentration of effective reducing agent(s) is essential. A thorough, routine workflow for 2DE involves reduction and alkylation prior to IEF (i.e., prior to the first dimension of separation), followed by a second reduction and alkylation step between the first and second dimensions (i.e., during equilibration, prior to SDS-PAGE) to most fully ensure that there are no intact disulfide bonds.

The reduction of disulfide bonds can be achieved by an equilibrium reaction with excess free thiols such as 2-mercaptoethanol (2-ME) or dithiothreitol (DTT) or in non-equilibrium reactions with trivalent phosphorus reagents such as tributylphosphine (TBP) [26,27,28,29]. A large excess of a sulfhydryl-reducing agent shifts the equilibrium of the oxidation/reduction reaction toward the fully reduced state. Historically, 2-ME was used at high concentrations (i.e., 200 mM) to ensure the maximal shift of the thiol–disulfide equilibrium toward the thiol form of the species [30,31,32]. However, considering the volatile and toxic nature of 2-ME, DTT has been widely adopted as an alternative reducing agent. The intramolecular, cyclic condensation during the oxidation of DTT drives the equilibrium toward the thiol-reduced state of the proteoform more efficiently compared to 2-ME, allowing for lower concentrations of DTT to be employed relative to 2-ME and further increasing the popularity and use of DTT [26,28]. However, DTT is a weak acid that consequently becomes negatively charged and can migrate toward the anode during IEF. Therefore, it is argued that DTT may not remain at constant concentrations in the basic region of an isolated pH gradient (IPG) strip, which could lead to the reformation of disulfide bonds and the possible precipitation of some disulfide-rich proteoforms [27,33]. As a result, minor artifacts, such as blurred spots and comet-like streaks, as well as the appearance or disappearance of spots, have been reported [24]. Thus, DTT has been supplemented with the non-ionic TBP that will not migrate to avoid the potential reformation of disulfide bonds during IEF. TBP thus helps to maintain reducing conditions throughout the IPG to give improved focusing and decreased streaking (i.e., better resolution) [11,18,26,27].

Alternative phosphines, such as TCEP, have been reported to be effective at lower concentrations due to their water-soluble, non-volatile, and stable characteristics [34,35,36]. Yet, despite being a ‘more water-soluble’ alternative to TBP, TCEP is a charged molecule and, thus, like DTT, can migrate within the IPG strip during IEF. Additionally, molecular modelling suggests that the thiolate of DTT is less sterically hindered than the phosphorus of TCEP due to steric crowding of the carboxyethyl substituents on TCEP and that the latter thus results in lower accessibility/reactivity with proteoform disulfides [36].

Additional organic disulfides have also been explored to provide a potential solution to the horizontal streaking that can arise in the basic region during the first dimension of 2DE. The streaking has been previously attributed to variations in the number of oxidized thiols in proteoforms, and the complete oxidation of thiol groups to mixed disulfides could thus eliminate streaking. Using an excess of 2-hydroxyethyl disulfide (HED, which has also come to be known as ‘DeStreak^TM^ reagent’) prior to the first dimension has been claimed to reduce streaking in the basic region; unfortunately, the comparisons were to quite low concentrations of DTT (i.e., notably well below excess) [37,38].

Thus, taking previous findings into consideration, an aspect of 2DE that has not undergone a thorough systematic analysis involves the reducing agents used, either alone or in combination, in the resolution of proteoforms. The present study thus contributes to the ongoing assessment and refinement of top–down proteomics by optimizing the concentration of reducing agents and thus, the proteoform resolution enabled by 2DE. To establish a protocol that is most likely to be broadly applicable (i.e., ‘universal’), we tested both mammalian and plant extracts for improved proteome resolution. Protein standards with known disulfide content, such as albumin, were also used to test for a quantitative relationship between DTT concentration and a number of disulfide bonds. As in previous studies, one-dimensional SDS-PAGE (1DE), the second dimension of 2DE, was first utilized to test reagents and optimize refinements, followed by implementation into a full 2DE protocol to quantitatively confirm and validate the findings [15,16,19,21]. Notably, improvements in the resolution of proteome extracts by 1DE, quantified by changes to the number of bands resolved and the intensity or resolution of a given band, likely represents effects on tens to hundreds of proteoforms per band [5,7,9,39].

The results indicate that, to date, many studies have been ‘under-powered’ in terms of proteoform reduction; optimizing the concentration and combination of reducing reagents significantly improves integrative 2DE-based top–down proteome analyses (and likely analyses by other methods as well).

## 2. Materials and Methods

All consumables were of ultra-pure or electrophoresis grade. Electrophoresis apparatuses, ReadyStrip^TM^ IPG Strips (7 cm, pH 3–10 non-linear), Bio-Lyte^TM^ carrier ampholytes, and Precision Plus Protein^TM^ Unstained Standards (10–250 kDa) were supplied by Bio-Rad Laboratories (Hercules, CA, USA). Isolated protein standards (Bovine Serum Albumin (BSA), Chicken Egg Albumin (CEA) and Chicken Egg Lysozyme (CEL)), and 2-hydroxyethyl disulfide (HED) were supplied by Sigma-Aldrich (St. Louis, MO, USA). Acrylamide/bis-acrylamide (40%, 37.5:1) solution, components of the protease inhibitor (PI) cocktail, thiourea, urea, *N,N,N’,N’*-tetramethylethylenediamine, sodium n-dodecyl sulfate, ammonium sulfate, glycerol, phosphoric acid, ammonium peroxydisulfate, and tributylphosphine (TBP) were obtained from Alfa Aesar (Haverhill, MA, USA). Sodium chloride, methanol, mineral oil, and dithiothreitol (DTT) were from Thermo Fischer Scientific (Waltham, MA, USA). Acrylamide and citric acid were obtained from BioShop Canada Inc (Burlington, ON, Canada). Coomassie Brilliant Blue G-250, CHAPS, tris hydrochloride, Tris-Glycine-SDS powder, and low-melting agarose were supplied by VWR (Radnor, PA, USA). Double glass-distilled water (ddH_2_O) was used throughout.

### 2.1. Sample Preparation

#### 2.1.1. Concentration and Purity Assessment of Isolated Protein Standards

Isolated protein standards (BSA, CEA, and CEL) were solubilized as previously described [16,19]. Initial measures of concentrations were according to the Beer–Lambert Law [16,19,40]. Gel-based purity analysis was carried out as previously described [15,41].

#### 2.1.2. Mammalian and Plant Proteome Sample Preparation

Previously snap-frozen whole mouse brains (*Mus musculus*) and green lentils (*Lens culinaris*) were homogenized via automated frozen disruption [13]. The resulting powdered samples were solubilized in 2X 1DE buffer (2X 1DB; 50 mM Tris [pH 8.8], 4% (*w*/*v*) SDS, 15% (*w*/*v*) glycerol, 0.002% (*w*/*v*) bromophenol blue and 1X PI). Aliquots of powdered total mouse brain and total green lentils were also solubilized in 2DE buffer (2DB; 8 M urea, 2 M thiourea, 4% *(w*/*v)* CHAPS, 1X PI). Total protein concentration was assessed using a solid-phase assay, as previously described [20].

### 2.2. 1DE: SDS-PAGE

SDS-PAGE was carried out as previously described with minor changes [19]. For 1DE, prior to SDS-PAGE, samples in 2X 1DB were thawed (once only) and diluted to a desired concentration (1 μg total protein loads per lane for protein standards (0.2 μg/μL) and 3 μg for extracted proteome samples (0.6 μg/uL); diluted to 1X 1DB), supplemented with reducing agent(s) (i.e., specific concentrations of DTT, TBP, and/or HED as indicated in all relevant figures), vortexed, and incubated for 20 min at RT (~22 °C) with intermittent gentle mixing. Following reduction, samples were vortexed, heated for 5 min at 100 °C using a dry heating block, sonicated for 5 min, and cooled to RT prior to loading in 2 mm or 5 mm wide wells in the stacking gel (5%T) over 1 mm thick 12.5%T resolving gels.

Following resolution, all gels were fixed in 1 M citric acid in 5% (*v*/*v*) acetic acid for 1 h at RT with gentle rocking [21]. Gels were then washed in ddH_2_O and stained in a colloidal Coomassie Brilliant Blue (cCBB) solution for 20 h with gentle rocking at RT [16,19,22]. Gels were subsequently destained with 0.5 M NaCl for 5 × 15 min prior to imaging by near-infrared fluorescence detection (nIRFD) using an Amersham Typhoon 5 Biomolecular Imager (Cytiva Life Sciences, Marlborough, MA, USA) with 685/≥750 nm excitation/emission, 50 μm pixel size, and PMT gain set to 600 V [6,19,22].

### 2.3. 2DE: IEF and SDS-PAGE

For 2DE, passive rehydration of IPG strips was carried out as previously described with some modifications [22]. Prior to rehydration, 100 μg extracted proteome (0.8 μg/μL) was reduced with either 12.5 mM DTT, 100 mM DTT + 5 mM TBP, or 100 mM HED at 25 °C for 1 h, followed by alkylation with 230 mM of acrylamide for 1 h. IEF was carried out at 17 °C, as previously described [21]. Prior to the second dimension, IPG strips were equilibrated with 6 M urea, 20% (*w*/*v*) glycerol, 2% (*w*/*v*) SDS, and 375 mM Tris [pH 8.8], first supplemented with either 130 mM DTT (the protocol previously used in our group, [6,11,19,21]), 12.5 mM DTT, 100 mM DTT + 5 mM TBP, or 100 mM HED for 10 min, then with 350 mM acrylamide for 10 min. SDS-PAGE was carried out as described in Section 2.2.

### 2.4. 1DE Image Analysis

All 1DE gel images were analyzed using Image Lab (v. 6.1.0, Bio-Rad Laboratories, Inc.) and ImageJ (v. 1.53, National Institutes of Health) [42]. For proteome extracts, in addition to the total number of resolved bands, well-resolved bands (i.e., visible, minimal streaking, no leading or trailing edges) over a range of molecular weights (MWs) were chosen for analysis of band intensity and full peak width at half-maximal height (FWHM). For protein standards, the main monomer band was chosen. Lane signal intensity and band intensity on gel images were analyzed using Image Lab, as previously described [6,21]. The ‘Lane Profile’ generated by Image Lab displays a cross-section of each lane, rotated 90 degrees to generate a chromatogram-like graph from the 3D display of the gel, with volume intensity on the *y*-axis and relative front (Rf) on the *x*-axis (Supplementary Figure S1 provides an example). Lane-based background subtraction was performed using a ‘rolling ball’ algorithm with the disk size set to 0.1 mm [43]. The total number of bands resolved was quantified as the total number of peaks on the chromatogram-like graph. A screen capture of the lane profile was taken with image size (1920 × 1080 pixels), and *y*-axis intervals were kept consistent between gels. To ‘calibrate’ the screen captures in ImageJ, the straight-line selection tool was used to create a horizontal straight line measuring the distance between 0.00 to 1.00 Rf and scaled to 1844 pixels. Maximal peak height was measured to calculate half maximal height using the straight-line selection tool; FWHM was then measured in pixels using the straight-line tool.

The resulting raw data for total lane signal, band intensity, and FWHM were processed in Excel 2019 (v. 16.52, Microsoft) and graphically displayed and statistically analyzed in Prism (v. 9.2.0, GraphPad Software). All conditions assessed were considered discreet treatments, and a one-way ANOVA with post-hoc Holm–Ŝídák pairwise comparisons were carried out [44]. Each experiment was independently replicated at least twice, and figure captions indicate sample size where *n* is the total number of 1DE sample lanes used for analyses; *p* values less than 0.05 were considered statistically significant. Statistically significant differences are indicated by ‘*’ where one symbol indicates *p* < 0.05, two indicate *p* < 0.005, and three indicate *p* < 0.001. Error is reported as standard error of the mean (SEM).

### 2.5. 2DE Image Analysis

2DE image analysis was carried out as previously described [6,22]. Using Image Lab, gel images were cropped to consistent dimensions, excluding the molecular weight (MW) markers and dye fronts, before analysis in Delta2D (v. 4.0.8, DECODON, Germany). Automated spot detection was completed on raw, unfused images to assess variance in individual gel spot count and on warped ‘union fused’ images to obtain absolute spot volumes. The resulting quantitation table yielded average normalized spot volumes across each reducing agent condition tested, quality of spot (i.e., spot circularity), coefficient of variation, and difference in protein abundance (*p* < 0.05, false discovery rate = 1%). Spot differences were considered legitimate when the relative standard deviation was ≤30% and increased or decreased fold change was ≥2. Error is reported as SEM.

## 3. Results

### 3.1. 1DE Experiments

To assess potential improvement in the resolution of proteoforms in 2DE, 1DE was first utilized to systematically test concentrations of reducing agents, individually and in combination. Potential improvements in the resolution were detected by changes in the total number of resolved bands, individual band intensity (i.e., signal), and FWHM. Proteome extracts resolved by 1DE yield relatively diffuse bands, each of which represents what would be numerous spots in 2DE gels and, therefore, countless proteoforms. Thus, decreases in FWHM (i.e., improved band resolution or ‘sharpness’) and increases in the intensity of 1DE bands serve as criteria to predict increasingly concentrated, compact spots in 2DE (i.e., increased resolution). Inter- and intra-gel total lane signals for all 1DE experiments were measured to ensure each lane was loaded with an equal amount of total protein (Supplementary Figure S2 given as an example) to ensure that changes in resolution could be attributed to varying reducing agent components, rather than spurious differences in lane loading [19,21]. No significant differences in lane loads were seen.

For simplicity and conciseness of data presentation, a single replicate (i.e., lane) of each condition is shown in the Results section, but all replicates and individual experiments with their complete respective gel images are available in supplementary data (Supplementary Figure S3).

#### 3.1.1. Initial Testing of DTT

**Figure 1:**
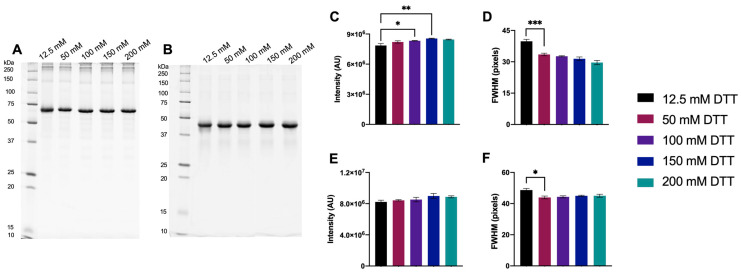
Protein Standards reduced with 12.5–200 mM DTT. Representative 1DE gel image of 1 μg BSA (**A**) and CEA (**B**) with bar graphs showing main monomer band intensity (**C** (BSA) and **E** (CEA)) and FWHM (**D** (BSA) and **F** (CEA)). Statistically significant differences are indicated by ‘*’ where one symbol indicates *p* < 0.05, two indicate *p* < 0.005, and three indicate *p* < 0.001 (One-way ANOVA, *n* = 4).

A broad range of DTT concentrations, 12.5–200 mM, was initially tested to identify an effective concentration. An effective concentration is defined as the minimum concentration required for DTT to be in assumed excess, driving the equilibrium of the proteome to the fully reduced state and above which no further significant improvements in resolution are detected. As 12.5 mM DTT was previously used routinely [16,19,20], this was selected as the baseline for comparison. To assess the relationship between disulfide bond content and DTT, protein standards with known disulfide bond content were used: BSA (17 disulfide bonds) and CEA (1 disulfide bond) (Figure 1; see also Supplementary Figure S4 for CEL, having 4 disulfide bonds). For BSA, there was a significant increase in monomer band intensity as DTT concentration was increased up to 150 mM, and FWHM was significantly lower with ≥50 mM DTT compared to 12.5 mM DTT. For CEA, no significant differences in monomer band intensity were measured, but FWHM significantly decreased with ≥50 mM compared to 12.5 mM DTT.

**Figure 2:**
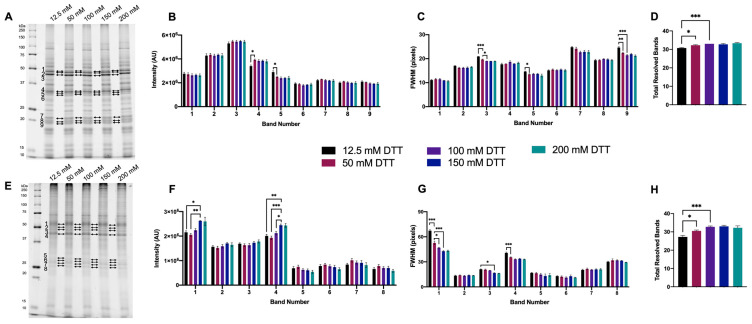
Proteome extracts reduced with 12.5–200 mM DTT. Representative 1DE gel lanes of total green lentil proteome extracts (**A**) and total mouse brain proteome extracts (**E**). Bar graphs showing band intensity (**B** (lentil) and **F** (brain)); FWHM (**C** (lentil) and **G** (brain)); total number of resolved bands (**D** (lentil) and **H** (brain)). Bands are as indicated on gel image. Statistically significant differences are indicated by ‘*’ where one symbol indicates *p* < 0.05, two indicate *p* < 0.005, and three indicate *p* < 0.001 (One-way ANOVA, *n* = 4).

For 1DE resolved extracted native proteomes, bands across a range of MWs were selected based on visually apparent qualitative improvements, as well as closely resolved bands. Most significant changes in the resolution of green lentil proteome extracts were detected as decreases in FWHM as DTT concentration was increased up to and above 100 mM (Figure 2A–C). Significant changes in the resolution of mouse brain proteome extracts were detected as increases in band intensity and decrease in FWHM as DTT concentration was increased up to and above 150 mM (Figure 2E–G). Treatment with ≥50 mM DTT yielded a significant increase in total resolved bands in both proteome extracts (Figure 2D,H).

Following these initial tests, 2 mm wide gel lanes were used to increase the number of lanes per gel, thus enabling an increased number of technical replicates to be resolved in parallel on a single gel. Additionally, narrow loading wells yield more concentrated 1DE-resolved bands that better represent 2DE-resolved spots, enabling better prediction of improved resolution in 2DE [19].

#### 3.1.2. Optimized DTT Concentration

As a further step to determine an optimal DTT concentration, a narrow range of concentrations were tested. Most significant improvements in the resolution were seen with either 100 mM or 150 mM DTT; thus, it was decided to test within the range of 100–160 mM DTT with 20 mM increments. No significant changes were detected in band intensity, FWHM, or the total number of resolved bands for BSA or proteome extracts between the different concentrations of DTT (gel images and detailed analyses outlined in supplementary data Figures S5 and S6). Overall, the minimally effective DTT concentration to be used in further experiments was thus defined as 100 mM.

#### 3.1.3. Supplementing DTT with TBP

To further optimize a reducing agent ‘cocktail,’ TBP was tested as an additional reducing agent to DTT. Our standard protocol utilizes 2.3 mM TBP in addition to DTT in the first step of reduction prior to IEF [11,19,21]; thus, 2.3 mM was tested in 1DE experiments, as well as half (1.15 mM) and double (4.6 mM) that concentration.

**Figure 3:**
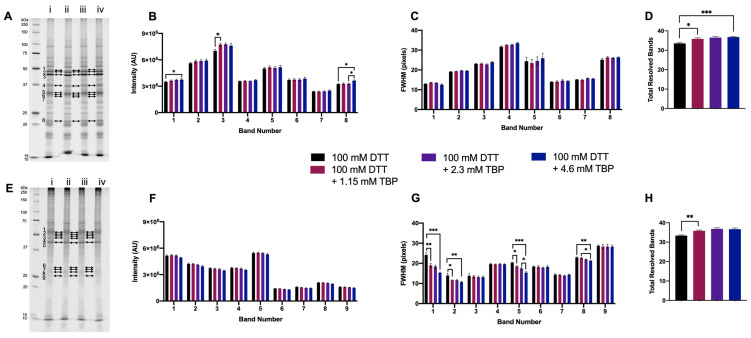
Proteome extracts reduced with 100 mM DTT and in combination with three concentrations of TBP. Representative 1DE gel lanes of total extracted green lentil proteome (**A**) and total extracted mouse brain proteome (**E**) with treatments of 100 mM DTT (i), 100 mM DTT + 1.15 mM TBP (ii), 100 mM DTT + 2.3 mM DTT (iii) and 100 mM DTT + 4.6 mM TBP (iv). Bar graphs showing band intensity (**B** (lentil) and **F** (brain)); FWHM (**C** (lentil) and **G** (brain)); total number of resolved bands (**D** (lentil) and **H** (brain). Bands are as indicated on gel image. Statistically significant differences are indicated by ‘*’ where one symbol indicates *p* < 0.05, two indicate *p* < 0.005, and three indicate *p* < 0.001 (One-way ANOVA, *n* = 6).

For green lentil proteome extracts, the intensity of two bands significantly increased with the addition of 4.6 mM TBP to 100 mM DTT. There were no significant changes in FWHM with the addition of TBP to green lentil proteome extracts (Figure 3A–C). With mouse brain proteome extracts, there were no significant differences in band intensity with the addition of TBP. However, for four bands, a significant decrease in FWHM was measured with the addition of 4.6 mM TBP (Figure 3E–G). Proteome extracts reduced with 100 mM DTT + ≥ 1.15 mM TBP yielded a significant increase in the total number of resolved bands (Figure 2D,H). Overall, 4.6 mM TBP was thus assessed as the optimal concentration to complement 100 mM DTT; for simplicity, this was increased to 5 mM for further experiments.

#### 3.1.4. Supplementing DTT with HED

**Figure 4:**
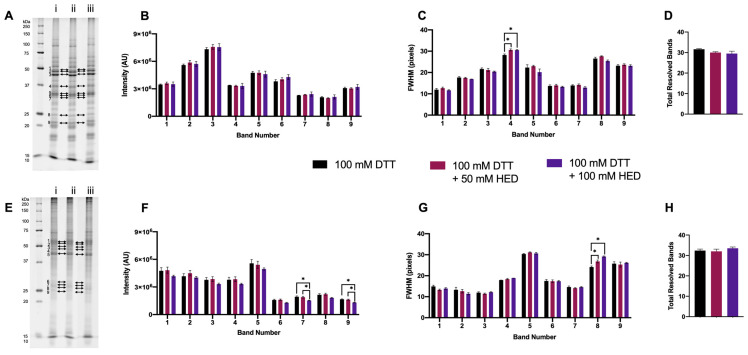
Proteome extracts reduced with 100 mM DTT and in combination with HED. Representative 1DE gel lanes of total extracted green lentil proteome (**A**) and total extracted mouse brain proteome (**E**) with treatments of 100 mM DTT (i), 100 mM DTT + 50 mM HED (ii), 100 mM DTT + 100 mM HED (iii). Bar graphs showing band intensity (**B** (lentil) and **F** (brain)); FWHM (**C** (lentil) and **G** (brain)); total number of resolved bands (**D** (lentil) and **H** (brain). Bands are as indicated on gel image. Statistically significant differences indicated by ‘*’ where *p* < 0.05 (One-way ANOVA, *n* = 4).

Two concentrations of HED were also tested as an adjunct to DTT, 100 mM—the indicated optimized concentration for DeStreak^TM^ and that previously reported to be in adequate excess—and 50 mM [37,45,46,47,48,49,50]. With the addition of HED to 100 mM DTT, there were no significant changes in the total number of resolved bands in either proteome extract (Figure 4D,H). For extracted green lentil proteome, there were also no changes measured in band intensity, although an increase in FWHM for a single band was seen with the addition of 50 mM and 100 mM HED compared to 100 mM DTT alone (Figure 4A–C). Significant changes to the resolution of the extracted mouse brain proteome with the addition of HED to DTT were measured as decreases in the intensity of two bands and a marked increase in FWHM for a single band in the lower MW region (Figure 4E–G). Thus, overall, supplementing DTT with HED tended to have a negative effect, if any, on proteome resolution.

#### 3.1.5. Final Testing of Reagents to Optimize for 2DE

**Figure 5:**
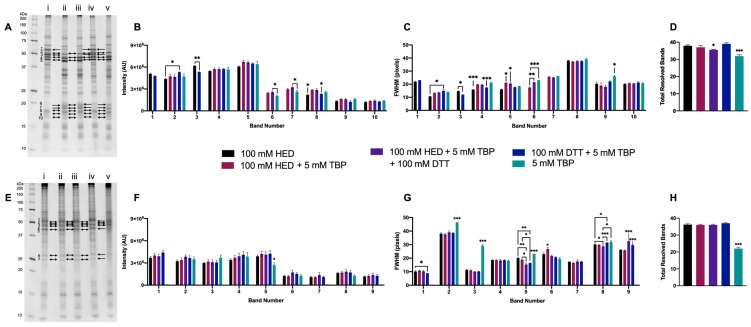
Proteome extracts reduced with DTT, TBP, and/or HED. Representative 1DE gel lanes of total extracted green lentil proteome (**A**) and total extracted mouse brain proteome (**E**) with treatments of 100 mM HED (i), 100 mM HED + 5 mM TBP (ii), 100 mM HED + 5 mM TBP + 100 mM HED (iii), 100 mM DTT + 5 mM TBP (iv), 5 mM TBP (v). Bar graphs showing band intensity (**B** (lentil) and **F** (brain)), FWHM (**C** (lentil) and **G** (brain)), and total number of resolved bands (**D** (lentil) and **H** (brain)). Bands are as indicated on gel image. Statistically significant differences are indicated by ‘*’ where one symbol indicates *p* < 0.05, two indicate *p* < 0.005, and three indicate *p* < 0.001 (One-way ANOVA, *n* = 6).

HED was also tested on its own as a single reducing reagent to evaluate previous claims that it reduced horizontal streaking in the basic region of 2D gels [37,46] and as an addition to DTT + TBP (i.e., combining all three reducing reagents). Similarly, TBP was also tested on its own and as an adjunct to HED (Figure 5).

Extracted green lentil proteome treated with 100 mM HED or 100 mM DTT + 5 mM TBP exhibited a notably different banding pattern relative to extracts treated with all three reagents, TBP only, or HED + TBP (Figure 5A). With the changes in banding patterns, green lentil proteome extracts treated only with 5 mM TBP, or treated with all three reagents, had a significantly lower number of resolved bands (Figure 5D). Significant increases in band intensity were detected with 100 mM DTT + 5 mM TBP (bands 3 and 8) or 100 mM HED (bands 2 and 8) (Figure 5B). The majority of significant decreases in FWHM were seen in samples treated with either 100 mM HED (bands 2, 4, and 5) or 100 mM DTT + 5 mM TBP (bands 3, 4, and 5). Treatment with HED + TBP and HED + TBP + DTT yielded a significant increase in FWHM for two bands (Figure 5C).

The banding patterns resolved with extracts of mouse brain proteome were largely consistent across treatments (Figure 5E), with the exception of 5 mM TBP alone, with which many resolved bands had a ‘shadowed’ or ‘streaked’ appearance, and this was quantitatively reflected in a decreased number of total resolved bands (Figure 5H). For one band, there was also a significant decrease in band intensity with 5 mM TBP (Figure 5F). Of the nine selected bands analyzed, only six were resolved with 5 mM TBP alone, and for each of these, FWHM was significantly increased. As with extracted green lentil proteome, decreased FWHM was found in those mouse brain proteome extracts treated with 100 mM HED (bands 8 and 9) or 100 mM DTT + 5 mM TBP (bands 2 and 5). Treatment with HED + TBP and HED + TBP + DTT yielded an increase in FWHM for bands 5 and 9 or no significant changes relative to HED alone or DTT + TBP (Figure 5G).

Extracted mouse brain and green lentil proteomes were also treated with 50 mM HED, alone and in combination with 5 mM TBP and 100 mM DTT, but no improvements were detected relative to treatment with 100 mM HED or 100 mM DTT + 5 mM TBP (Supplementary Figure S3).

Overall, the majority of significant improvements in 1DE resolution were seen with 100 mM DTT + 5 mM TBP, and to some extent also with 100 mM HED, in both proteome samples; TBP alone proved to be largely unsatisfactory, particularly in terms of total resolved bands (Figure 5D,H).

### 3.2. Testing in the Full 2DE Protocol

To evaluate the improvement of proteoform resolution in 2DE, we assessed total signal, total spot count, and changes in average normalized spot volumes (i.e., signal) and spot quality (i.e., spot circularity). Significant increases in total signal, total spot count, and normalized spot volumes were considered improvements. Spot quality, as defined in Delta2D, is a measure from 0 to 1 of how similar a spot is to a perfect gaussian distributed shape; thus, values close to 1 indicate very high spot quality. Again, for simplicity and conciseness of data presentation, a single gel replicate of each condition is shown in the Results section, but all replicates are available in supplementary data (Supplementary Figures S7–S9).

**Figure 6:**
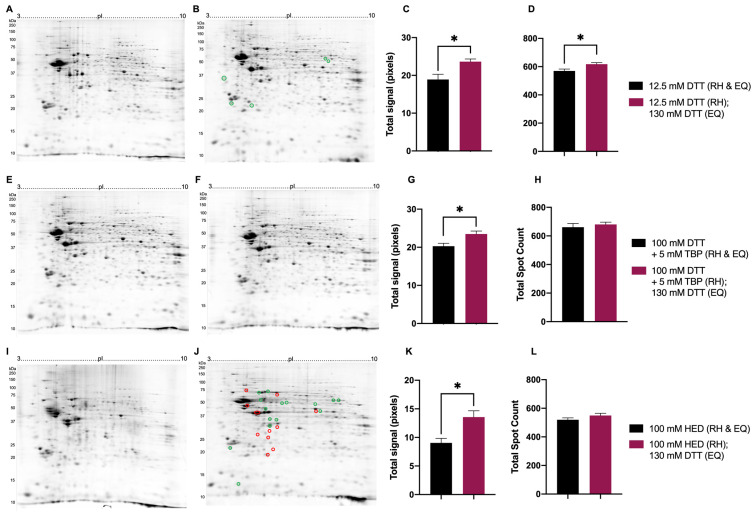
Representative 2DE gel images of resolved total mouse brain proteome comparing treatments prior to rehydration (RH) and during equilibration (EQ). Extracted mouse brain proteome treated with 12.5 mM DTT (RH and EQ) (**A**), 12.5 mM DTT (RH) + 130 mM DTT (EQ) (**B**), 100 mM DTT + 5 mM TBP (RH and EQ) (**E**), 100 mM DTT + 5 mM TBP (RH) + 130 mM DTT (EQ) (**F**), 100 mM HED (RH and EQ) (**I**), 100 mM HED (RH) + 130 mM DTT (EQ) (**J**). Bar graphs showing total signal (**C** corresponding to images **A** and **B**, **G** corresponding to images **E** and **F**, **K** corresponding to images **I** and **J**) and total spot count (**D** corresponding to images **A** and **B**, **H** corresponding to images **E** and **F**, **L** corresponding to images **I** and **J**). Green and red circles identify significant differences in average normalized spot volumes when fold change was ≥2 (green = increased; red = decreased) Statistically significant differences are indicated by ‘*’ where *p* < 0.05 (*t*-test, *n* = 6).

To determine the treatment yielding optimal resolution in 2DE, samples treated with either 12.5 mM DTT (the ‘baseline’ standard), 100 mM DTT + 5 mM TBP, or 100 mM HED before and after IEF (i.e., before rehydration and during equilibration) were compared to samples treated with either 12.5 mM DTT, 100 mM DTT + 5 mM TBP, or 100 mM HED before IEF and with 130 mM DTT after IEF (the latter being the current standard) (Figure 6).

Mouse brain proteome extracts reduced with 130 mM DTT during equilibration (EQ) yielded an increase in total signal (Figure 6C,G,K). Compared to the reduction with 12.5 mM DTT during EQ, the total spot count also significantly increased in mouse brain proteome extracts reduced with 130 mM DTT during EQ (Figure 6D). When treated with either 100 mM DTT + 5 mM TBP or 100 mM HED prior to rehydration (RH), no significant changes were detected in total spot count between different reduction treatments during EQ (Figure 6H,L). Regarding individual spot intensities of extracted mouse brain proteomes treated with 12.5 mM DTT prior to RH, those reduced with 130 mM DTT during EQ yielded four spots with increased absolute spot volumes (≥2-fold change) (Figure 6A,B). With respect to treatment with DTT + TBP prior to RH, no significant changes in absolute spot volumes were detected between different reduction treatments during EQ (Figure 6E,F). Mouse brain proteome extracts reduced with 100 mM HED prior to RH and 130 mM DTT during EQ yielded 15 spots with significantly increased absolute spot volumes compared to the reduction with 100 mM HED prior to RH and during EQ (Figure 6I–J).

Overall, the reducing agent treatments to be used in final analyses and comparisons to determine a protocol that yields an optimal resolution of proteome extracts in 2DE were as follows: for reduction prior rehydration, 12.5 mM DTT (as the ‘baseline’ comparative standard), 100 mM DTT + 5 mM TBP, or 100 mM HED, and each treated with 130 mM DTT during equilibration between the first and second dimensions of resolution.

**Figure 7:**
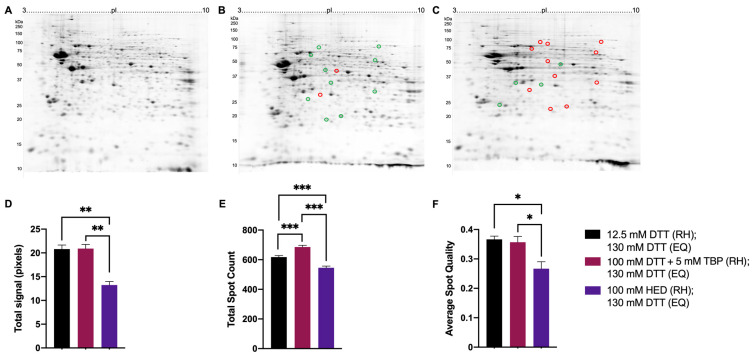
Representative 2DE gel images of resolved total mouse brain proteome. Extracted mouse brain proteome with treatments of 12.5 mM DTT (**A**), 100 mM DTT + 5 mM TBP (**B**), 100 mM HED (**C**) prior to rehydration and each treated with 130 mM DTT during equilibration (i.e., the current standard). Bar graphs showing total signal (**D**), total spot count (**E**), and average spot circularity (**F**). Green and red circles identify significant differences in average normalized spot volumes relative to reduction with 12.5 mM DTT when fold change was ≥2 (green = increased; red = decreased). Statistically significant differences are indicated by ‘*’ where one symbol indicates *p* < 0.05, two indicate *p* < 0.005, and three indicate *p* < 0.001 (One-way ANOVA, *n* = 9).

In mouse brain proteome extracts, total signal and average spot quality were significantly lower when treated with 100 mM HED (13.23 ± 0.74 pixels; 0.26 ± 0.02) compared to treatment with either 12.5 mM DTT (20.79 ± 0.86 pixels; 0.37 ± 0.01) or 100 mM DTT + 5 mM TBP (20.91 ± 0.87 pixels; 0.36 ± 0.02). Reduction with DTT + TBP yielded the highest spot count (689 ± 11 spots; Figure 7B,E). Relative to reduction with 12.5 mM DTT, changes in spot abundances were detected over a range of MW and pI, with 11 spots significantly lower in abundance when reduced with 100 mM HED and 10 spots significantly increased in abundance when reduced with 100 mM DTT + 5 mM TBP. Qualitatively, exposure to HED also yielded the poorest quality 2DE gels (i.e., lowest proteome resolution).

**Figure 8:**
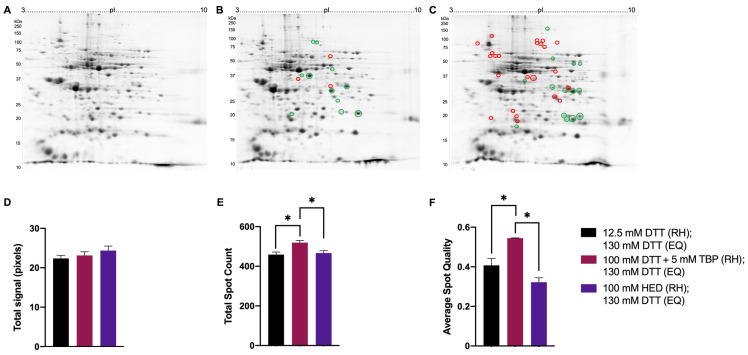
Representative 2DE gel images of total green lentil proteome. Extracted green lentil proteome with treatments of 12.5 mM DTT (A), 100 mM DTT + 5 mM TBP (B), 100 mM HED (**C**) prior to rehydration and each treated with 130 mM DTT during equilibration. Bar graphs showing total signal (**D**), total spot count (**E**), and average spot circularity (**F**). Green and red circles identify significant differences in average normalized spot volumes relative to reduction with 12.5 mM DTT when fold change was ≥2 (green = increased; red = decreased). Statistically significant differences are indicated by ‘*’ where *p* < 0.05 (One-way ANOVA, *n* = 2–3).

Green lentil proteome extracts treated with 100 mM DTT + 5 mM TBP also had significantly higher total spot counts and average spot quality (519 ± 11 spots; 0.54 ± 0.01) compared to treatment with 12.5 mM DTT (459 ± 13 spots; 0.41 ± 0.03) and 100 mM HED (467 ± 11 spots; 0.32 ± 0.04) (Figure 8E,F). There were no significant changes in total signal across different treatments (Figure 8D). Relative to reduction with 12.5 mM DTT, 11 spots significantly increased in abundance when reduced with 100 mM DTT + 5 mM TBP, and 23 spots significantly decreased when reduced with 100 mM HED (Figure 8A-C).

## 4. Discussion

Although there have been previous reports assessing, to some extent, the use of reducing reagents in 2DE, there does not appear to be a direct assessment between different sample types treated with these reagents, either alone or in combination [27,35,37,38,46,51,52]. It is acknowledged that there will not be a ‘perfect’ one-size-fits-all method; however, the goal here was to establish an optimized general protocol to enhance 2DE resolution and thus a breadth of research supported by routine integrative top–down proteomic analyses [7,8,9,11,12,53,54,55,56,57,58,59,60,61]. The data clearly indicate that for native proteome extracts, reduction using 100 mM DTT + 5 mM TBP prior to sample rehydration into IPG strips is superior to the current ‘standard’ as well as to other reagents previously reported in the literature. The current standard of 130 mM DTT (e.g., [11]) during the equilibration between the first and second dimensions of 2DE proved superior to the other reagents/combinations tested. Thus, in contrast to some opinions [33], it is also apparent that reduction (and alkylation) is best carried out both before and after IEF.

Since the adoption of DTT as an alternative to 2-ME, it has become a standard reagent for reducing disulfide bonds in many 1DE and 2DE protocols. However, despite its popularity, an appropriate, effective concentration to ensure a complete reduction of proteoforms in native proteomes, particularly during IEF, appeared not to have previously been quantitatively established. As with many routine aspects of 2DE, and indeed many analytical protocols, this was another which seemed somewhat ‘historically’ based on whichever earlier protocol was inherited or adopted from the literature; thus, concentrations generally in the range of 1–100 mM have been widely employed [7,8,19,24,62,63,64,65]. To determine an effective concentration, we first aimed to test for a relationship between disulfide bond content and DTT concentration using commercially purified protein standards. Initially, it was hypothesized that a higher number of disulfide bonds would require an increased concentration of DTT. This was demonstrated in the comparison of BSA (17 disulfide bonds) to CEA (1 disulfide bond), in which higher DTT concentrations were required for significant improvements in resolving BSA. Conversely, we also resolved chicken egg lysozyme (4 disulfide bonds; Figure S4), but no significant improvements were detected with increasing DTT concentrations. This was consistent with previous reports that DTT should be used in excess to ensure complete reduction (i.e., shift the equilibrium to the fully reduced state), regardless of disulfide bond content [26,28]. Furthermore, an effective concentration may not simply correlate with the number of disulfide bonds but rather also with reagent accessibility to disulfide bridges and perhaps additionally with the nature of amino acid domains surrounding the cysteines involved in the bridges. Furthermore, these prove to be critical concepts when resolving extracted native proteomes, as the number and accessibility of disulfide bonds are inevitably unknown. Thus, following the reduction of extracted proteomes with a range of DTT concentrations, the 1DE data indicated that 100 mM DTT was the minimal effective concentration needed for optimal analysis. Lower concentrations of DTT were not sufficient, essentially ‘underpowering’ the analysis to varying extents.

Due to the weak acid and ionic character of DTT, TBP has been used to supplement DTT, specifically during the first dimension of 2DE. TBP reduces thiols in a stoichiometric reaction and, therefore, can be utilized at lower concentrations. TBP has been demonstrated in previous reports to improve resolution, indicated by minimized horizontal streaking and increased spot count [27]. Although TBP was not tested here on its own in 2DE, mouse brain and green lentil proteome extracts reduced with TBP alone yielded poorer resolution in 1DE, with fewer resolved bands compared to DTT supplemented with TBP.

Previous reports have claimed the poor resolution and inconsistent spot patterns in the basic region of 2DE proteome maps are a result of incomplete reduction and inter- and intra-chain disulfide bonds reforming near the cathode during IEF. The reduction mechanism of 2-ME gave rise to the idea that the oxidation of thiol groups to mixed disulfides may prevent reoxidation, and thus, HED was adopted based on claimed remarkable improvements in resolution, notably decreased ‘streaking’ and tailed spots [37,46,47,48,49,50]. The results here differ markedly from those earlier reports in that proteome extracts treated with DTT + TBP yielded significantly higher spot counts and better overall spot quality (i.e., less streaked, tailed spots). The likely explanation for such striking differences is that the earlier reports compared high HED concentrations (i.e., 100 mM) to completely inadequate concentrations of DTT (i.e., below excess or an established effective concentration, thus resulting in an incomplete reduction of disulfides) [37,48,50]. Certainly, a careful review of the original report of reduced streaking using HED reveals gel images (apparently single replicates) that were only evaluated qualitatively [37]. The data here (Figure 6, Figure 7 and Figure 8) indicate that even the lowest concentration of DTT is largely superior in terms of ensuring the reduction of native proteome extracts and does not support the notion that reduction with HED results in the lower occurrence of streaking in the basic region; indeed, if anything, the qualitative assessment suggests slightly increased streaking in the mid pI range when HED is used. The unsatisfactory results with HED may be attributed to the reduction mechanism relative to that of DTT. The reduction of disulfide bridges by DTT produces a thermodynamically favoured six-membered ring that drives the equilibrium towards the reduced state [26,28] and, thus, may be more efficient at reducing disulfide bridges in proteoforms and ensuring complete reduction compared to HED. Previous reports have demonstrated intact disulfide bonds result in a more compact conformation of a proteoform, resulting in changes to electrophoretic mobility and SDS-binding [66]. The binding of SDS during the equilibration step in 2DE aids in proteoform solubilization and facilitates the transfer of proteoforms from the IPG strip into the SDS-resolving gel in the second dimension, thus providing a likely explanation for the much lower total signal and spot counts detected in proteome extracts reduced with HED compared to DTT.

One potential limitation in our study is the selection of bands for the assessment of resolution in 1DE. Although reasonable attempts were made for a complete, thorough assessment, qualitative (i.e., visually apparent) improvements in resolution guided band selection for subsequent quantitative analysis. However, this method of selection may be limited in assessing changes in band intensity or FWHM. Nonetheless, as with our previous work to improve integrative top–down proteomic analyses, testing a range of conditions first using 1DE enabled efficient and effective narrowing down of conditions to finally be vetted by 2DE. This represents a significant saving of time and resources. Secondly, this study focused primarily on the reduction of proteomes during the rehydration and equilibration steps of 2DE. It might also prove useful to test these reagents in the solubilization buffer used for proteome extraction to assess potential improvements in proteoform solubilization, resolution, and, thus, depth of proteome analyses. While comparable systematic analyses of alkylating agents might also be suggested, excellent work indicates that the excess acrylamide used here is quite likely the best available treatment [67,68].

To address the full complexity of proteomes, that is, routinely analyzing the constituent proteoforms beyond simply cataloguing canonical amino acid sequences, we must implement deep, quantitative analyses having the necessary analytical power and rigour [1,2,3,5]. Currently, this is best (and routinely) implemented using integrative top–down proteomic analyses that fully utilize the complementary strengths of 2DE and LC/TMS [1,2,4,5,6,7,8,9,10,11,18,19,22,69]. In contrast to bottom–up analyses, which infer the identity of only canonical protein sequences or mass-spectrometry-intensive top–down approaches that, while sensitive and thorough, can generally only handle species of <20–30 kDA, integrative top–down analyses using 2DE as the front-end of resolution enable deep proteomic analyses at the necessary level of proteoforms. The data presented in this study, along with decades of significant refinements and improvements, enable a genuine understanding of the complexity and diversity of the proteome by increasing analytical rigor [2,4,6,7,8,9,10,18].

## 5. Conclusions

Reducing extracts of native proteomes with 100 mM DTT + 5 mM TBP prior to the first dimension of separation yielded improved resolution and detection by 2DE relative to other reagents previously used in comparable studies. We acknowledge there is not a ‘perfect’ one-size-fits-all method; however, the goal here was to establish an optimized general protocol to enhance 2DE resolution and, thus, routine integrative top–down proteomic analyses. Further optimization of these conditions is encouraged for the analysis of different native proteomes, and the ‘optimized’ reduction protocol established here is strongly suggested as a critical starting point to ensure the deepest possible quantitative proteome analyses, regardless of the analytical techniques employed. As with our previous work, we encourage further testing, refinement, and optimization even of ‘established’ protocols, again, regardless of the analytical techniques employed. Only such rigorous and routine assessments will ensure the truest possible proteome analyses as required to identify critical changes to proteoforms.

## Data Availability

The data presented in this study are available in the Supplementary Material.

## Supplementary Materials

The following supporting information can be downloaded at: https://www.mdpi.com/article/10.3390/proteomes11010010/s1; Figure S1: Representative chromatogram via lane profile of total green lentil proteome extracts with 100 mM DTT; Figure S2: Representative analysis of total lane signal to confirm equivalent total protein loads per lane; Figure S3: 1DE Gel Images; Figure S4: Chicken egg lysozyme reduced with 12.5–200 mM DTT; Figure S5: BSA reduced with 100–160 mM DTT; Figure S6: Proteome extracts reduced with 100–160 mM DTT; Figure S7: Gel images of resolved mouse brain proteome extracts; Figure S8: Gel images of resolved mouse brain proteome extracts; Figure S9: Gel images of resolved green lentil proteome extracts.
