# Supplementary material for: Optimized Proteome Reduction for Integrative Top–Down Proteomics
Source: Proteomes. 2023 Mar 6;11(1):10. doi: 10.3390/proteomes11010010 (PMC10059017; doi:10.3390/proteomes11010010)
Supplement: Supplementary file 1 [file proteomes-11-00010-s001.zip › proteomes-2200194-supplementary.pdf]

Supplementary Data

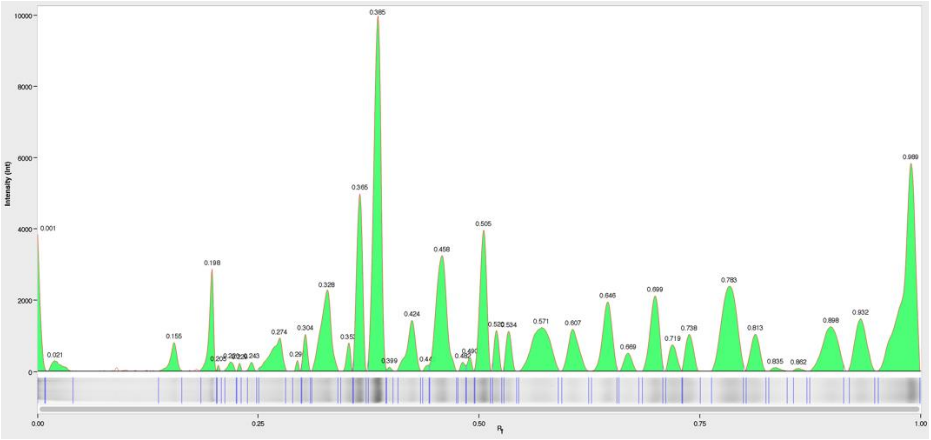

Figure S1. Representative chromatogram via lane profile of total green lentil proteome extracts reduced with 100mM DTT.

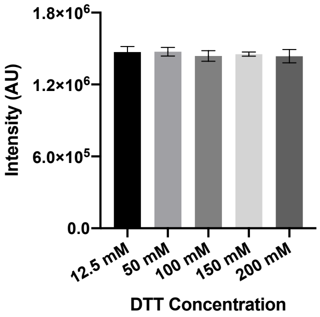

Figure S2. Representative analysis of total lane signal to confirm equivalent total protein loads per lane (from initial testing of DTT with total mouse brain proteome extracts (Figure 2 in main article)).

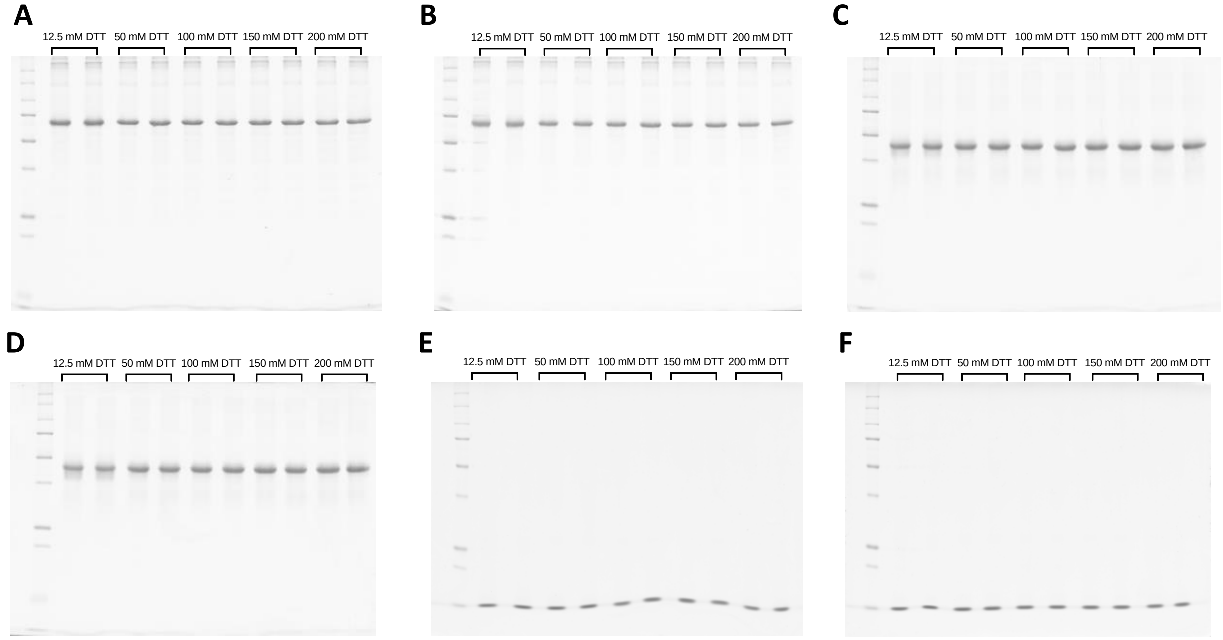

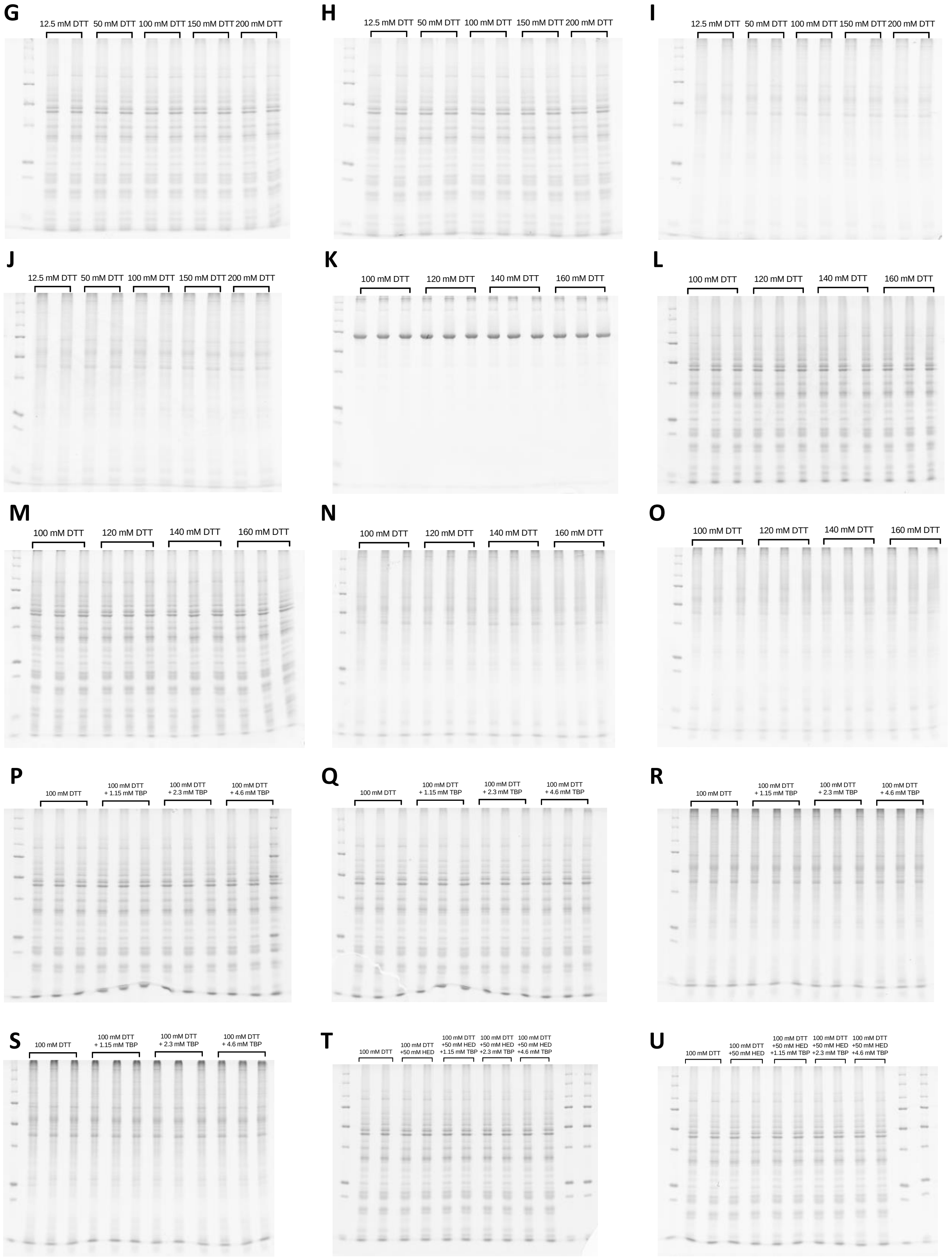
G
12.5 mM DTT 50 mM DTT 100 mM DTT 150 mM DTT 200 mM DTT
H
12.5 mM DTT 50 mM DTT 100 mM DTT 150 mM DTT 200 mM DTT
I
12.5 mM DTT 50 mM DTT 100 mM DTT 150 mM DTT 200 mM DTT
J
12.5 mM DTT 50 mM DTT 100 mM DTT 150 mM DTT 200 mM DTT
K
100 mM DTT 120 mM DTT 140 mM DTT 160 mM DTT
L
100 mM DTT 120 mM DTT 140 mM DTT 160 mM DTT
M
100 mM DTT 120 mM DTT 140 mM DTT 160 mM DTT
N
100 mM DTT 120 mM DTT 140 mM DTT 160 mM DTT
O
100 mM DTT 120 mM DTT 140 mM DTT 160 mM DTT
P
100 mM DTT
100 mM DTT + 1.15 mM TBP
100 mM DTT + 2.3 mM TBP
100 mM DTT + 4.6 mM TBP
Q
100 mM DTT
100 mM DTT + 1.15 mM TBP
100 mM DTT + 2.3 mM TBP
100 mM DTT + 4.6 mM TBP
R
100 mM DTT
100 mM DTT + 1.15 mM TBP
100 mM DTT + 2.3 mM TBP
100 mM DTT + 4.6 mM TBP
S
100 mM DTT
100 mM DTT + 1.15 mM TBP
100 mM DTT + 2.3 mM TBP
100 mM DTT + 4.6 mM TBP
T
100 mM DTT
100 mM DTT +50 mM HED
100 mM DTT +50 mM HED +1.15 mM TBP
100 mM DTT +50 mM HED +2.3 mM TBP
100 mM DTT +50 mM HED +4.6 mM TBP
U
100 mM DTT
100 mM DTT +50 mM HED
100 mM DTT +50 mM HED +1.15 mM TBP
100 mM DTT +50 mM HED +2.3 mM TBP
100 mM DTT +50 mM HED +4.6 mM TBP

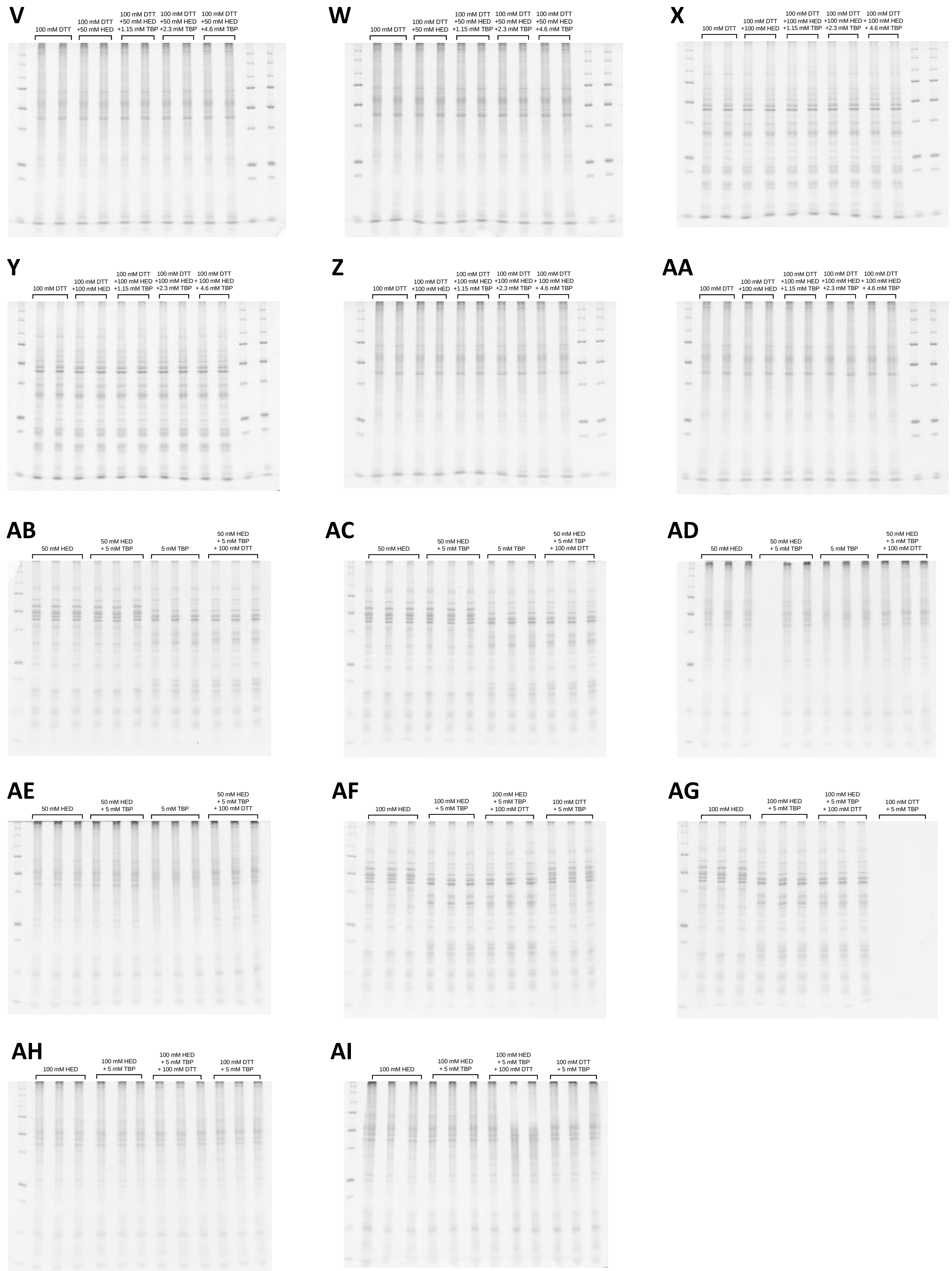

Figure S3. 1DE Gel Images. All raw 1DE gel images used for image analysis. BSA (**A**, **B**, **K**); CEA (**C**, **D**); CEL (**E, F**); total green lentil proteome extracts (**G**, **H**, **L**, **M**, **P**, **Q**, **T**, **U**, **X**, **Y**, **AB**, **AC**, **AF**, **AG**); total mouse brain proteome extracts (**I**, **J**, **N**, **O**, **R**, **S**, **V**, **W**, **Z**, **AA**, **AD**, **AE**, **AH**, **AI**).

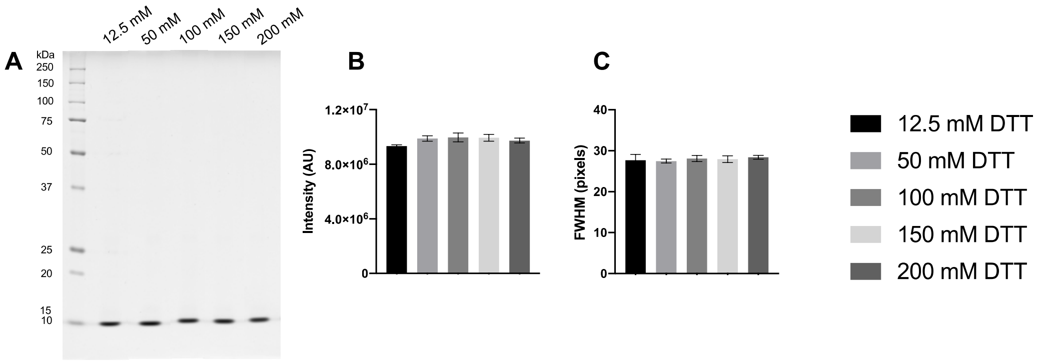

Figure S4. Chicken egg lysozyme reduced with 12.5-200 mM DTT. Representative 1DE gel image of CEL in 5 mm wells with bar graphs showing main monomer band intensity (**B**) and FWHM (**C**). (One-way ANOVA, n=4).

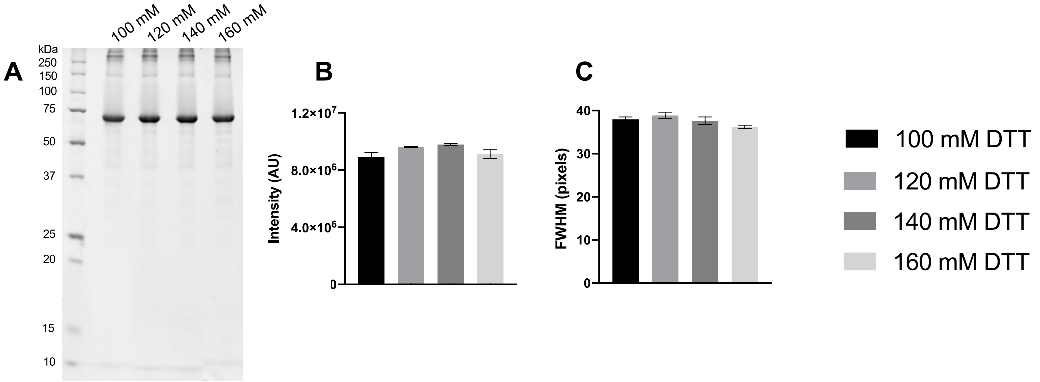

Figure S5. BSA reduced with 100-160 mM DTT. Representative 1DE gel image of BSA with bar graphs showing main monomer band intensity (**B**) and FWHM (**C**). (One-way ANOVA, n=3).

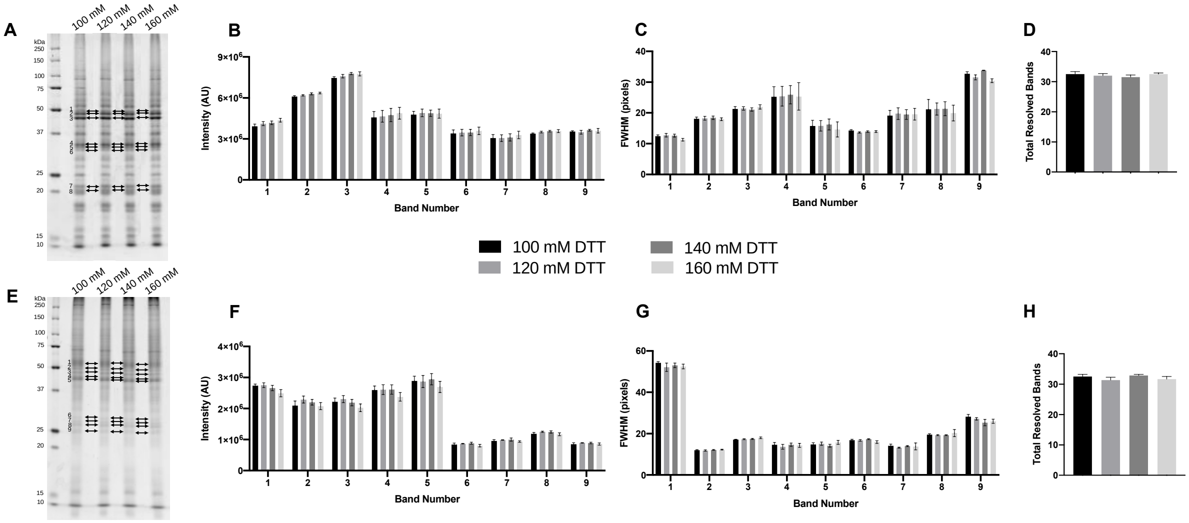

Figure S6. Proteome extracts reduced with 100-160 mM DTT. Representative 1DE gel lanes of total green lentil proteome extracts (**A**) and total mouse brain proteome extracts (**E**). Bar graphs showing band intensity (**B** (lentil) & **F** (brain));FWHM (**C** (lentil) & **G** (brain)); total number of resolved bands (**D** (lentil) & **H** (brain)). Band numbers are as indicated on gel image. (One-way ANOVA, n=6).

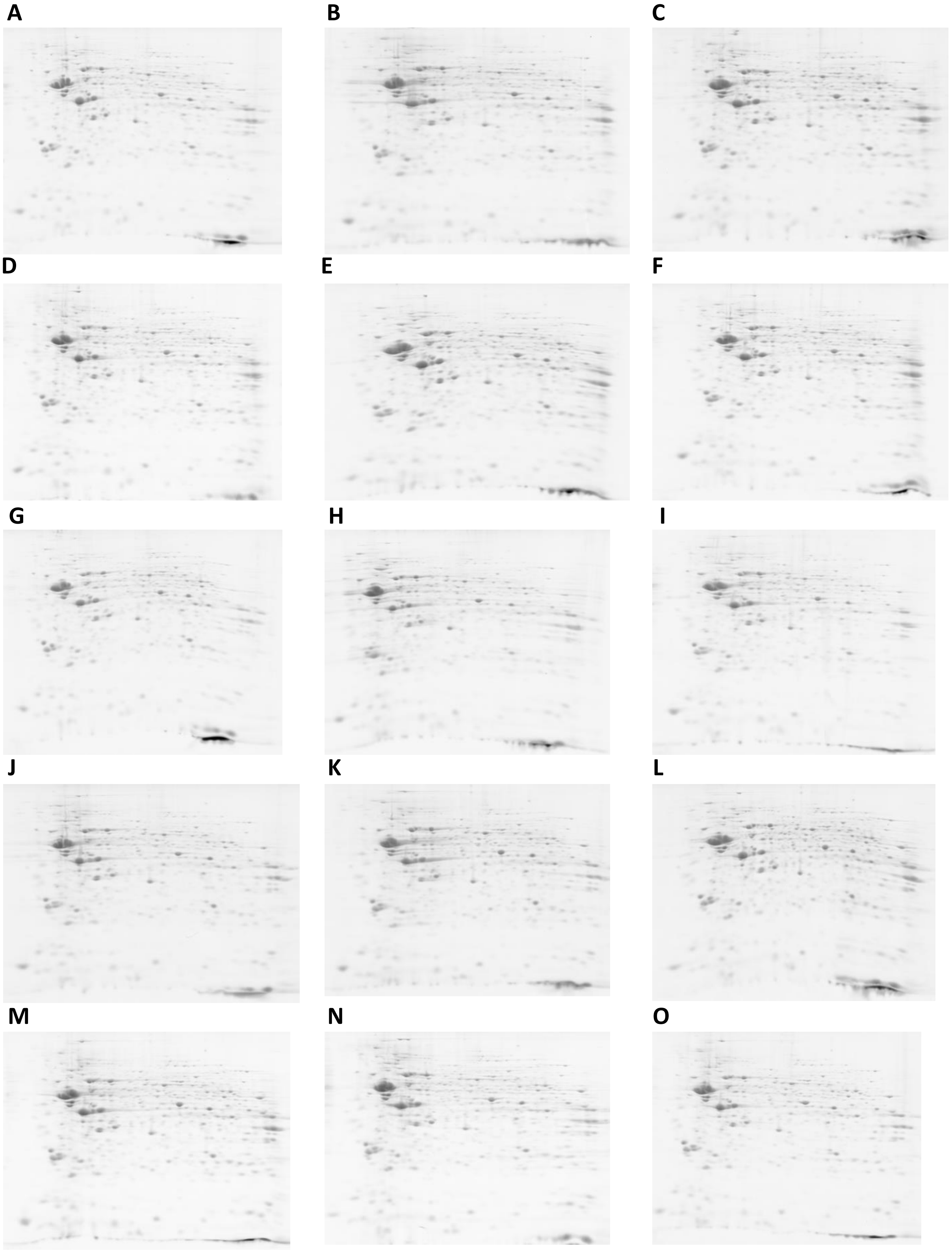
A
B
C
D
E
F
G
H
I
J
K
L
M
N
O

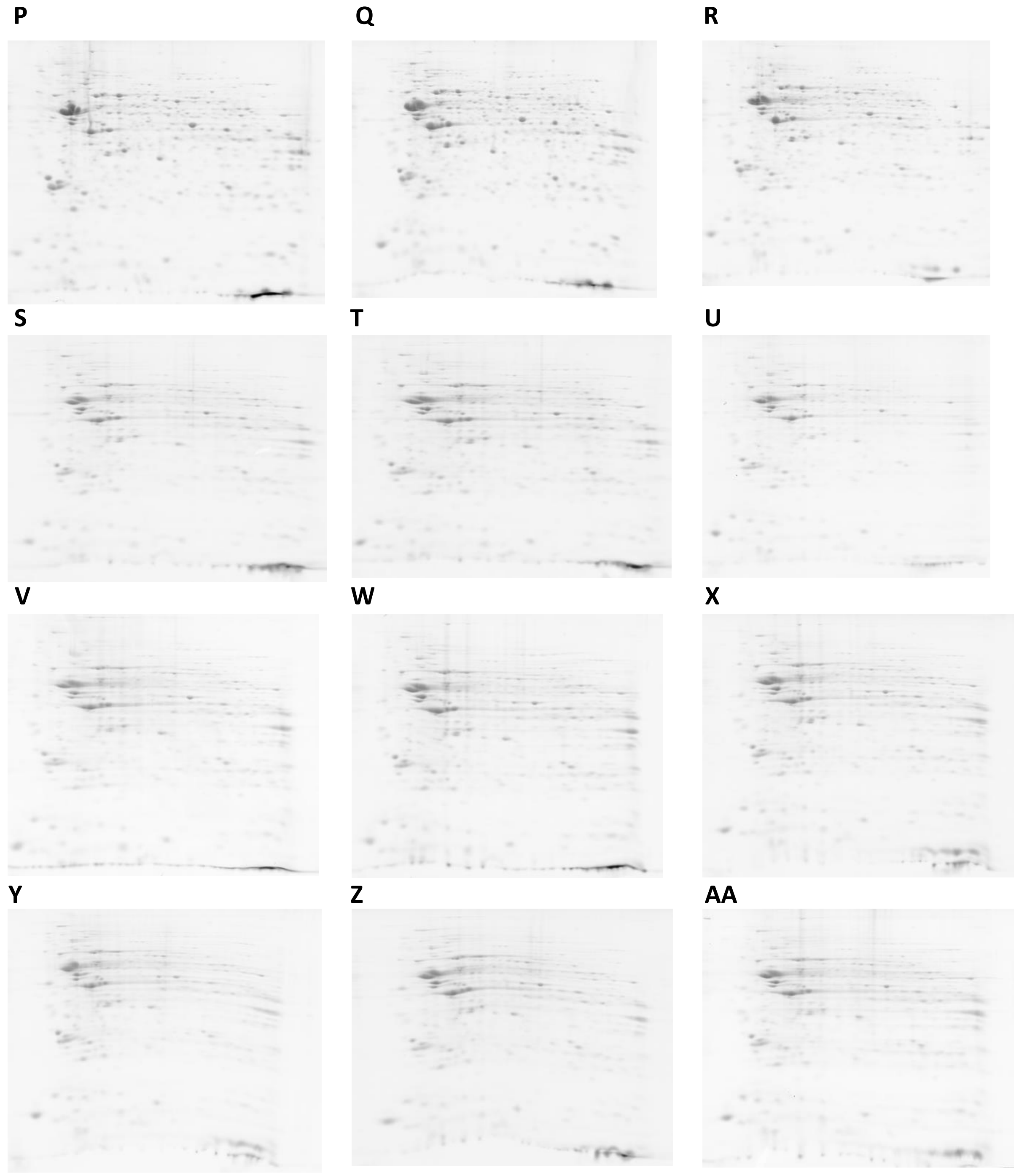

Figure S7. Gel images of resolved mouse brain proteome extracts. Raw 2DE gel images of resolve used for image analysis. Proteome extracts reduced with 12.5 mM DTT (**A-I**); 100 mM DTT +5 mM TBP (**J**-**R**); 100 mM HED (**S**-**AA**) prior to rehydration. All samples were then reduced with 130 mM DTT during equilibration.

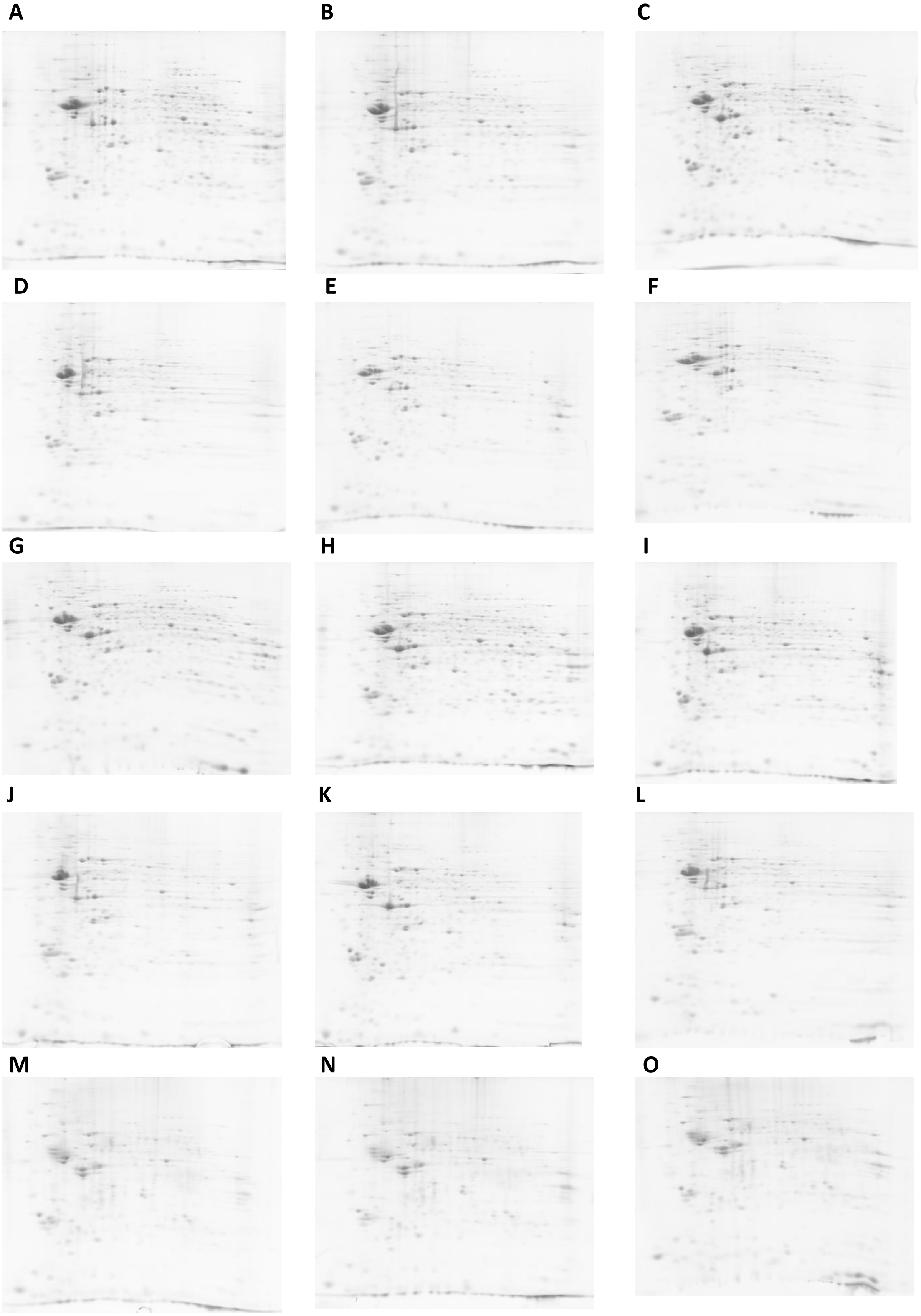
A
B
C
D
E
F
G
H
I
J
K
L
M
N
O

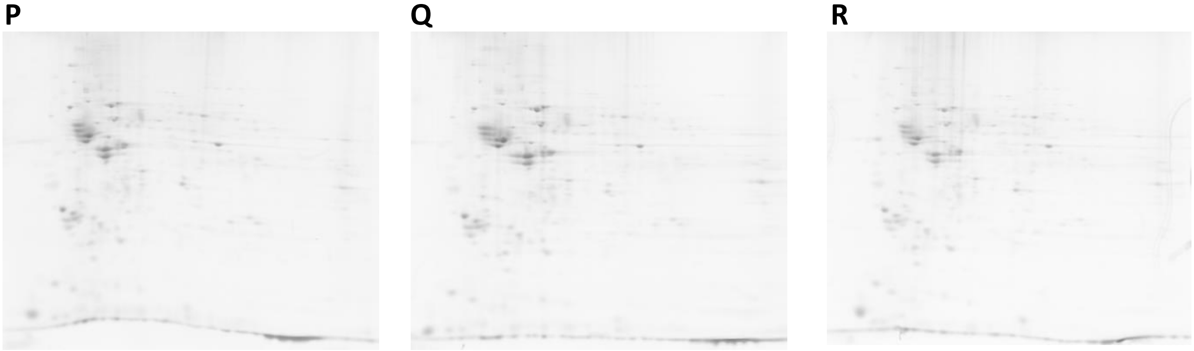

Figure S8. Gel images of resolved mouse brain proteome extracts. Raw 2DE gel images used for image analysis. Proteome extracts reduced with 12.5 mM DTT (**A**-**F**); 100 mM DTT +5 mM TBP (**G**-**L**); 100 mM HED (**M**-**R**) prior to rehydration and during equilibration.

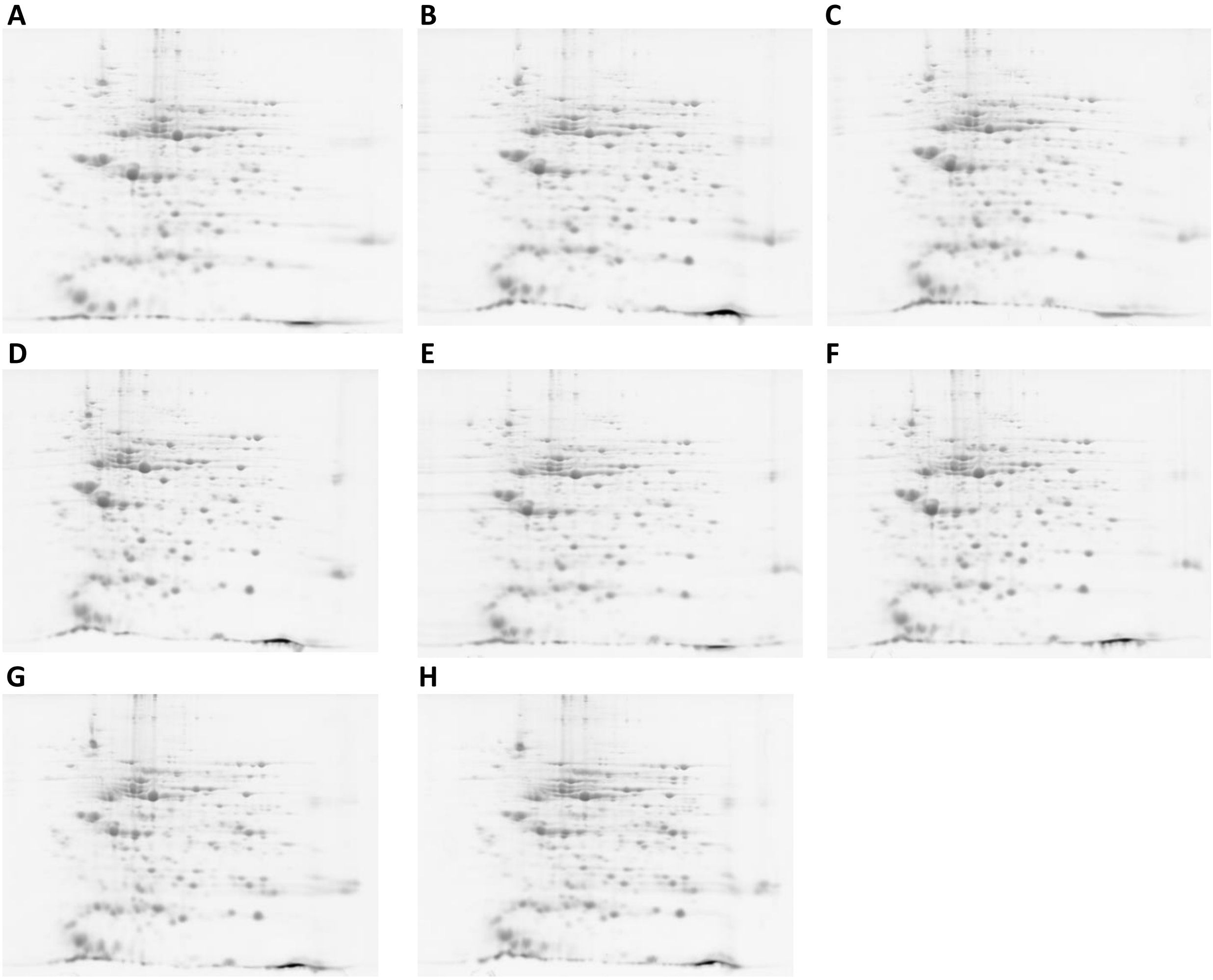

Figure S9. Gel images of resolved green lentil proteome extracts. Raw 2DE gel images used for image analysis. Proteome extracts reduced with 12.5 mM DTT (**A**-**C**); 100 mM DTT +5 mM TBP (**D**-**F**); 100 mM HED (**G**-**H**) prior to rehydration. All samples were reduced with 130 mM DTT during equilibration.
